# Daily loneliness and subjective well-being as a function of older adults’ sexual orientation

**DOI:** 10.1093/geronb/gbag131

**Published:** 2026-07-02

**Authors:** Geva Shenkman, Amit Shrira, Yuval Shaia, Adi Poritzky Doga, Yuval Palgi

**Affiliations:** Dina Recanati School of Medicine, Reichman University (IDC Herzliya), Herzliya, Israel; Department of Social & Health Sciences, Faculty of Social Sciences, Bar-Ilan University, Ramat Gan, Israel; Baruch Ivcher School of Psychology, Reichman University (IDC Herzliya), Herzliya, Israel; Baruch Ivcher School of Psychology, Reichman University (IDC Herzliya), Herzliya, Israel; Department of Gerontology, Faculty of Social Welfare and Health Sciences, University of Haifa, Haifa, Israel; (Psychological Sciences Section)

**Keywords:** Positive affect, Negative affect, Happiness, Daily diary, Sexual minority populations

## Abstract

**Objectives:**

Although loneliness has been consistently linked to poorer subjective well-being (SWB) in later life, little is known about how this association fluctuates daily or whether it varies as a function of sexual orientation. Drawing on evidence regarding the cumulative effects of prolonged stigma exposure and intersecting identities in later life, the present study examines whether the daily coupling between loneliness and SWB differs between older lesbian, gay, and bisexual (LGB) and heterosexual adults, due to the double jeopardy of age- and sexual minority related stigmas.

**Methods:**

Community-dwelling lesbian, gay, bisexual, and heterosexual middle-aged and older adults (*N *= 151, *M*_age_ = 59.73, range = 50–79) completed measures of daily loneliness and SWB indicated by happiness, positive affect, and negative affect over 14 consecutive days.

**Results:**

Older LGB respondents reported greater negative affect, as well as lower happiness, compared with their heterosexual counterparts. Additionally, on days characterized by higher loneliness, participants reported lower daily happiness and positive affect and higher negative affect. These day-to-day associations were stronger among older LGB than heterosexual respondents, although no such moderating effect was observed for positive affect.

**Discussion:**

Examining day-to-day variations in loneliness and SWB among older adults across sexual orientation groups is pivotal. From an applied perspective, this line of research can help identify older minority populations that may be especially susceptible to the detrimental emotional consequences of daily loneliness.

Loneliness is a distressing state reflecting a discrepancy between the quality and quantity of social relationships individuals desire and those they perceive they have (Lee et al., 2021). It is a pressing public health concern (World Health Organization [WHO], 2023) linked to poorer health, morbidity, and mortality (Beutel et al., 2017; Wang et al., 2018), as well as lower subjective well-being (SWB), indexed by happiness, positive affect (PA), and negative affect (NA) (Diener et al., 1999), among both older heterosexual and older lesbian, gay, and bisexual (LGB) populations (Fish & Weis, 2019; Torres et al., 2024). However, the daily association between loneliness and SWB and its moderation by sexual orientation remains unexamined. Accordingly, this study examines sexual orientation differences in the daily association between loneliness and SWB among older adults. Beyond advancing theoretical knowledge, this investigation also aims to determine whether older LGB adults exhibit greater affective reactivity to daily loneliness, thereby informing tailored interventions.

Compared to heterosexual counterparts, older LGB adults are at heightened risk for loneliness, partly due to a lower likelihood of being partnered or having children (Hsieh & Liu, 2021), and for lower SWB (Grabovac et al., 2019). These vulnerabilities are attributed to chronic and cumulative stressors related to stigma, prejudice, and discrimination, as articulated in Meyer’s minority stress model (Meyer, 2003). Many older LGB individuals may have come of age when concealing their sexual orientation was common, and exposure to homophobia may have been frequent, with lasting effects on SWB (Detwiler et al., 2023; Fredriksen-Goldsen, 2023). In Israel, historical criminalization of homosexuality and ongoing structural inequality (e.g., lack of same-sex marriage), alongside dominant religious norms that prohibit homosexuality, may undermine well-being in older LGB adults (Shenkman, 2024) and contribute to isolation and loneliness (Eppler-Hattab et al., 2026). Complementing this framework, the Iridescent Life Course perspective (Fredriksen-Goldsen et al., 2019) suggests that intersecting identities (e.g., sexual minority status and aging) may heighten vulnerability to loneliness and poorer SWB in later life.

Although loneliness has been assessed using brief and longitudinal measures (Hughes et al., 2004; Rodeiro et al., 2025), daily assessments remain largely understudied in older minority populations (Institute of Medicine, 2011). Addressing this gap may reveal heightened day-to-day affective vulnerability to loneliness among older LGB adults, a population in which such reactivity may reflect the cumulative effects of stigma exposure and intersecting identities in later life.

Building on prior research showing that loneliness is associated with lower SWB among both older heterosexual and LGB adults (Fish & Weis, 2019; Torres et al., 2024), and grounded in minority stress theory (Meyer, 2003) and the Iridescent Life Course perspective (Fredriksen-Goldsen et al., 2019), we expected two group differences: (1) differences between LGB and heterosexual individuals in mean levels of SWB and loneliness (i.e., lower SWB and higher loneliness in LGB); and (2) differences in the daily sensitivity to loneliness (i.e., stronger daily coupling between loneliness and SWB in LGB). Accordingly, we hypothesized that: (1) older LGB adults, compared with their heterosexual counterparts, would report higher levels of loneliness, and poorer SWB, as reflected in lower happiness and PA and higher NA; (2) on days characterized by greater loneliness, individuals would report lower happiness and PA and higher NA; and (3) these daily associations between loneliness and SWB would be stronger among LGB than heterosexual participants.

## Method

### Participants and procedure

The sample comprised 151 Israeli adults aged 50–79 (*M*age = 59.73, standard deviation [*SD*] = 6.89) recruited via convenience sampling, including 78 heterosexual participants (48 women, 30 men) and 73 LGB participants (37 women, 36 men). The LGB group included 32 lesbian women, 35 gay men, five bisexual women, and one bisexual man.

Data were collected between November 19, 2024 and February 5, 2025. Participants first completed an online baseline questionnaire assessing background characteristics, followed by daily evening surveys over 14 consecutive days. Recruitment involved targeted outreach to older sexual minority communities (e.g., LGBTQ+ events) and advertisements on social media and online platforms (Facebook, WhatsApp), as well as recruitment of heterosexual participants via the same platforms and student networks. Eligibility criteria included being aged 50 or older and having proficiency in Hebrew. The survey link was distributed via Qualtrics by students who also provided daily reminders and technical support.

The analytic sample was restricted to participants who provided complete responses to at least eight of the 14 daily assessments, to ensure reliable within-person estimates (cf. Kornadt et al., 2026). On average, participants completed 13.01 daily surveys, yielding a total of 1,965 observations. The study received Institutional Review Board approval from Reichman University (P_2024181). All participants provided informed consent, and no identifying information was collected to ensure anonymity.

### Measures

Daily loneliness was assessed using the Three-Item Loneliness Scale (Hughes et al., 2004). Participants rated their loneliness on a three-point scale (1 = *hardly ever* to 3 = *often)*. Scores ranged from 3 to 9, with higher scores indicating greater loneliness. Internal consistency was good (mean α = 0.86, *SD* = 0.04, range = 0.74 to 0.89).

Daily happiness was assessed using the Happiness single-item scale (Abdel-Khalek, 2006). Participants answered the single item (“How happy have you felt in the last 24 hours”) and rated their daily happiness on an 11-point scale (0–10). In this study, the single item had a temporal stability of 0.73.

Daily negative and positive affect were assessed using 12 items (six NA, six PA) from the National Survey of Midlife Development in the United States (Mroczek & Kolarz, 1998; Hebrew version Palgi et al., 2021). Participants rated emotions (e.g., depressed, cheerful) on a 5-point scale (1 = ‘*none of the time’* to 5 = ‘*all of the time’*). The mean score was computed, with higher values indicating greater NA/PA. Internal consistency was good (mean_NA_  *α* = 0.83, *SD* = 0.04, range = 0.76 to 0.89; mean_PA_  *α* = 0.92, *SD* = 0.03, range = 0.86 to 0.95).

#### Background characteristics

Sexual orientation was assessed by self-identification (heterosexual, lesbian, gay, bisexual, or another identity). Participants also reported sociodemographic characteristics, including age, gender, education (1 = elementary to 7 = graduate degree/PhD), relationship status (0 = not partnered, 1 = partnered), parenthood, self-rated health (0 = low to 4 = high), and perceived financial status (0 = very poor to 4 = very good).

### Data analysis

To examine group differences between older LGB and heterosexual adults in loneliness and SWB indicators, we initially conducted analyses of covariance (ANCOVAs). Next, multilevel modeling was employed to account for the nested structure of the data, with daily observations (Level 1) nested within individuals (Level 2). This approach is appropriate for repeated daily assessments, as it accounts for the non-independence of observations within persons and allows examination of both within- and between-person effects. Daily loneliness and SWB (NA, PA, and happiness) were modeled at Level 1, whereas sexual orientation (heterosexual vs LGB) was modeled at Level 2. Models were estimated with random intercepts using restricted maximum likelihood (REML). Daily loneliness was person-mean-centered and was entered alongside participants’ mean loneliness across the assessment period. Sexual orientation was dummy-coded. Cross-level interactions tested whether sexual orientation moderated the within-person association between daily loneliness and SWB. Fixed effects included daily loneliness, sexual orientation, and their interaction. Random effects included intercepts, allowing baseline levels of SWB to vary across individuals. All analyses adjusted for age, gender, educational attainment, and self-rated financial status and health, as these factors have been consistently linked to SWB (DeNeve & Cooper, 1998). Models were estimated in SPSS 28; interactions were probed using the procedures outlined by Preacher et al. (2006). Additional model specifications and equations are provided in the Supplementary Material.

Statistical power was evaluated using the EMAtools package. Assuming approximately 150 participants across 14 days and an intraclass correlation (ICC) of 0.40 (Bodner et al., 2021), the study was sufficiently powered to detect medium-sized effects (*d* > 0.50), with power exceeding 90% and approaching 99% under full compliance.

## Results

### Preliminary analyses

Descriptive analysis showed that most participants held an academic degree (90.1%), were partnered (89.4%), had children (83.4%), reported above-moderate finances (69.5%), and had good/very good health (82.8%). LGB and heterosexual participants did not differ on most sociodemographics (*p*s = .058–.53). However, the groups differed in educational attainment, partnership status, and parenthood (*p*s < .001), with LGB participants more likely to hold academic degrees (98.6% vs 82.1%) and less likely to be partnered (80.8% vs 97.4%) or have children (69.9% vs 96.2%).

Table 1 presents the means, standard deviations, and bivariate between-person correlations for the main study variables. Heterosexual and LGB individuals did not differ in PA (*p* = .29), but significantly differed in loneliness, NA, and happiness (*p* ranged from .011 to .035). At the between-person level, loneliness was negatively correlated with PA (*r *= −0.27, *p* < .001) and happiness (*r *= −0.21, *p* = .010), and positively correlated with NA (*r* = 0.55, *p* < .001). All SWB indicators were significantly correlated with each other (*p* < .001). Daily NA, PA, happiness, and loneliness showed a considerable amount of within-person variance as reflected by their 1-ICCs (NA = 0.36, PA = 0.31, happiness = 0.27, and loneliness = 0.33).

**Table 1:** Descriptive statistics for the study variables.

Variables	Full sample		Heterosexual	LGB	1	2	3	4
	*M*/% (*SD*)	Range	*M*/% (*SD*)	*M*/% (*SD*)				
**1. Average daily negative affect**	1.45 (0.45)	1–5	1.39 (0.40)	1.52 (0.50)	—			
**2. Average daily positive affect**	3.37 (0.68)	1–5	3.40 (0.61)	3.34 (0.75)	−0.50***	—		
**3. Average daily happiness**	6.97 (1.98))	0–10	7.32 (1.65)	6.59 (2.24)	−0.45***	0.64***	—	
**4. Average daily loneliness**	3.68 (1.08)	3–9	3.53 (0.86)	3.85 (1.25)	0.55***	−0.27***	−0.21**	—
**6. Sexual orientation (LGB)**	48.3	—	—	—	0.15	−0.05	−0.19*	0.15
**7. Chronological age**	59.73 (6.89)	50–79	58.79 (6.23)	60.73 (7.44)	−0.06	0.12	0.01	0.11
**8. Gender (woman)**	56.3	—	61.5	50.7	0.07	−0.10	0.03	0.11
**9. Education level (academic degree)**	90.1	—	82.1	98.6	−0.16*	0.17*	−0.02	−0.20*
**10. Relationship status (married/with partner)**	89.4	—	97.4	80.8	−0.21**	0.10	0.29***	−0.14
**11. Children (yes)**	83.4	—	96.2	69.9	−0.15	−0.07	0.07	−0.15
**12. Self-rated financial status**	2.92 (0.86)	0–4	3.05 (0.75)	2.78 (0.96)	−0.19*	0.13	0.05	−0.35***
**13. Self-rated health**	3.18 (0.79)	0–4	3.22 (0.69)	3.14 (0.88)	−0.32***	0.41***	0.39***	−0.32***

*Note.* LGB = lesbian, gay, and bisexual. Correlations represent between-person correlations for the full sample based on individual mean values for the daily variables.

* *p* <.05.

** *p* <.01.

*** *p* <.001.

To test Hypothesis 1, we conducted a series of ANCOVAs with group (heterosexual versus LGB individuals) as the independent variable, NA, PA, and happiness as the dependent variables, and age, gender, education level, self-rated financial status, and self-rated health as covariates. The results indicated significant differences between groups in NA (*F*[1, 144] = 4.96, *p* = .028, partial *η*^2^ = 0.033) and happiness (*F*[1, 144] = 4.58, *p* = .034, partial *η*^2^ = 0.031), such that LGB individuals reported higher levels of NA and lower levels of happiness compared to their heterosexual counterparts. However, there were no significant differences in loneliness, although the effect approached significance (*F*[1, 144] = 3.72, *p* = .056, partial *η*^2^ = 0.025); nor in PA (*F*[1, 144] = 1.14, *p* = .288, partial *η*^2^ = 0.008).

### Multilevel models

To test Hypotheses 2 and 3, we conducted multilevel models with random intercepts to account for between-person variability. Table 2 presents the multilevel models predicting daily SWB by daily loneliness, sexual orientation, and their interaction. Daily loneliness had significant effects on SWB. There was a significant interaction between loneliness and sexual orientation in predicting NA, such that the daily coupling between loneliness and NA was stronger among LGB (estimator = 0.11, *p* < .001) relative to heterosexual individuals (estimator = 0.09, *p* < .001). Furthermore, there was a significant interaction between loneliness and sexual orientation in predicting happiness, such that the daily coupling between loneliness and happiness was stronger among LGB (estimator = −0.38, *p* < .001) relative to heterosexual individuals (estimator = −0.29, *p* = .009). The interaction term between loneliness and sexual orientation was not significant when predicting PA. Notably, in multilevel analyses controlling for relationship and parenthood status, the significant interaction effect between loneliness and sexual orientation predicting NA and happiness remained significant. In the primary analyses, we refrained from controlling for relationship and parenthood status, as LGB status is highly correlated with access to marriage and parenthood. However, we added relationship and parenthood status to additional models to test whether LGB status had a net effect (or an interaction with loneliness) on SWB, beyond these socio-demographics.

**Table 2:** Unstandardized coefficients of multilevel models predicting negative affect, positive affect, and happiness.

Predicting daily SWB by	Negative affect	Positive affect	Happiness
**Fixed effects**			
Intercept, γ_00_	1.61 (0.37)	2.85 (0.60)	5.86 (1.56)
Sexual orientation (LGB), γ_01_	−0.05 (0.10)	−0.26 (0.14)	−0.22 (0.35)
Chronological age, γ_02_	−0.01 (0.00)	0.02* (0.01)	0.02 (0.02)
Gender (woman), γ_03_	0.01 (0.06)	−0.05 (0.10)	0.07 (0.26)
Education level, γ_04_	−0.13 (0.11)	0.19 (0.18)	0.29 (0.46)
Self-rated financial status, γ_05_	0.02 (0.04)	0.02 (0.06)	−0.19 (0.16)
Self-rated health, γ_06_	−0.10* (0.04)	0.13 (0.07)	0.58** (0.17)
Average loneliness, γ_07_	0.11*** (0.03)	−0.11* (0.05)	−0.04 (0.14)
Daily loneliness, γ_10_	0.06*** (0.02)	−0.13*** (0.02)	−0.23*** (0.05)
Sexual orientation*daily loneliness, γ_11_	0.04* (0.02)	0.01 (0.03)	−0.14* (0.06)
**Random effects**			
Between-person, *u*_0_	0.13*** (0.02)	0.34*** (0.04)	20.30*** (0.27)
Within-person, *e*	0.11*** (0.00)	0.20*** (0.01)	10.00*** (0.03)
**Pseudo-*R^2^* within-person**	0.04	0.04	0.05
**Pseudo-*R^2^* between-person**	0.36	0.28	0.19

*Note*. LGB = lesbian, gay, and bisexual; SWB = subjective well-being. *Standard errors* are reported in parentheses. Pseudo-*R^2^* within- and between-person is calculated based on Raudenbush and Bryk (2002).

* *p* < .05.

** *p* < .01.

*** *p* < .001.

## Discussion

The present study contributes to the literature on loneliness and minority stress by examining day-to-day associations between loneliness and SWB across sexual orientation groups in later life. Older LGB adults reported higher NA and lower happiness than heterosexual adults, with no group differences in PA and a marginal difference in loneliness. Supporting Hypothesis 2, higher daily loneliness was associated with lower happiness and PA and higher NA. In line with Hypothesis 3, these associations were moderated by sexual orientation, with stronger daily links between loneliness and happiness and NA among mid-life and older LGB adults, indicating heightened vulnerability.

Higher NA and lower happiness among LGB adults align with prior research documenting poorer SWB in this population (Grabovac et al., 2019; Hsieh & Liu, 2021), commonly attributed to the cumulative and chronic effects of stigma, prejudice, and discrimination across the life course (Frost & Meyer, 2023).

While prior studies have linked loneliness to poorer SWB in both LGB and heterosexual adults using global assessments (Fish & Weis, 2019; Torres et al., 2024), the present study extends this work by showing day-to-day associations, which, except for PA, were stronger among older LGB adults. These findings align with minority stress theory and the Iridescent Life Course perspective, highlighting how prolonged stigma and intersecting identities heighten daily emotional vulnerability in older sexual minority adults (Fredriksen-Goldsen et al., 2019). This may also help explain the stronger daily loneliness-SWB linkage observed in this group, reflecting this heightened vulnerability. At the same time, strengths-based perspectives such as crisis competence (Friend, 1991) suggest that lifelong coping with stigma may foster resilience and adaptive coping, and the balance between vulnerability and resilience in explaining these daily patterns warrants further investigation.

Interestingly, the absence of group differences in PA and of sexual-orientation moderation in its daily link with loneliness may reflect conceptual distinctions between PA and happiness, which represent different components of well-being (Schick et al., 2023). Happiness is typically assessed globally and tied to social connectedness, whereas PA includes both high- and low-arousal states that may relate differently to loneliness. Future research should further examine these differential associations.

When interpreting the present study’s findings, it is important to consider the sociocultural context of older LGB adults in Israel, as outlined in the Introduction. In Israel, historical criminalization of homosexuality, lingering stigma, and dominant religious norms prohibiting homosexuality may heighten daily vulnerability to loneliness and lower SWB among older LGB adults (Shenkman, 2024).

Several limitations of the present study should be acknowledged. First, a convenience sample limits generalizability. Second, the sexual minority group included only LGB participants; future research should also include transgender and non-binary older adults, as it may shed light on a unique “minority within a minority” experience, potentially characterized by heightened vulnerability to the effects of loneliness on SWB. Due to limited subgroup sizes, LGB participants were analyzed as a single group, and the groups differed in gender composition, with unequal representation of women. Future studies would benefit from examining daily loneliness and SWB separately across sexual minority subgroups, as experiences may differ between gay men and lesbian women, with potentially stronger loneliness-SWB associations among gay men, given their comparatively lower accumulated relational resources across the life course (Cohn-Schwartz et al., 2025). As the present study assessed general loneliness, future research could distinguish general loneliness from loneliness related to specific social ties, such as sexual minority community connectedness. In addition, minority stress-related experiences (e.g., homophobia or hate-related experiences; perceived social rejection and social anxiety) were not directly measured and therefore could not be examined in relation to loneliness or SWB, nor controlled for in the analyses. Finally, daily reports may have been shaped by unmeasured day-specific events, and data were collected during a period of war-related stress in Israel, which may have influenced responses. Future research should examine whether these patterns replicate in more stable periods.

Despite these limitations, this study highlights the importance of examining daily loneliness and its implications for SWB across sexual orientation groups in later life. Findings indicate heightened emotional vulnerability to daily loneliness among older LGB adults, underscoring the need for clinicians to monitor loneliness and follow affirmative wellness counseling guidelines (Chaney & Whitman, 2020). For researchers, the results support studying loneliness as a day-to-day process rather than a static condition. At the policy level, healthcare organizations should develop loneliness-prevention initiatives tailored to older sexual minority populations, given links between structural inequality, loneliness, poorer health outcomes, and reduced longevity.

## Supplementary Material

gbag131_Supplementary_Data

## Data Availability

Data, analytic methods, and materials will be made available to other researchers upon request to the first author. The current study was not preregistered.
