# Supplementary material for: Daily loneliness and subjective well-being as a function of older adults’ sexual orientation
Source: J Gerontol B Psychol Sci Soc Sci. 2026 Jul 2;81(9):gbag131. doi: 10.1093/geronb/gbag131 (PMC13481675; doi:10.1093/geronb/gbag131)
Supplement: Supplementary file 1 [file gbag131_supplementary_data.pdf]

The Journals of Gerontology, Series B: Psychological Sciences and Social Sciences
Supplementary Material: Shenkman et al. Daily loneliness and subjective well-being as a function of older adults' sexual orientation.

Data Analysis

The Level 1 (within-person) was parametrized as:

$$SWB_{it} = \beta_{0i} + \beta_{1i}(\text{Loneliness}_{it}) + e_{it}. \quad (1)$$

In this model, SWB (Happiness; Positive affect; Negative affect) for person *i* at time *t*, SWB_{it}, is a function of (a) an intercept parameter for person *i* (across *t* times for which the person provided data), β_{0i}; (b) a slope parameter that captures the coupling effect of loneliness for person *i*, β_{1i}; and (c) the within-individual random error, e_{it}.

The level 2 (between-person) model was parameterized as:

$$\beta_{0i} = \gamma_{00} + \gamma_{01}(\text{Sexual orientation}_i) + \gamma_{02}(\text{Chronological age}_i) + \gamma_{03}(\text{Gender}_i) + \gamma_{04}(\text{Education level}_i) + \gamma_{05}(\text{Self-rated financial status}_i) + \gamma_{06}(\text{Self-rated health}_i) + \gamma_{07}(\text{Average loneliness}_i) + u_{0i}. \quad (2)$$

$$\beta_{1i} = \gamma_{10} + \gamma_{11}(\text{Sexual orientation}_i) \quad (3)$$

in this model, γ_{00} represents the intercept of SWB for a person with sexual orientation=0 (i.e., heterosexual); γ_{01} represents the difference in SWB between heterosexual and LGB individuals (for a person with loneliness=0); γ_{02} to γ_{07} represent the effects of covariates and the between-person level average loneliness (across the two weeks of the study) on SWB, and u_{0i} represents the error of β_{0i}. Additionally, γ_{10} represents the slope of the effect of loneliness for a person with sexual orientation=0, and γ_{11} represents the effect of sexual orientation on the daily coupling of loneliness and SWB.

Main Analyses

Table 1 (in main text) presents means, *SD*, and bivariate between-person correlations for the study variables. The groups did not differ in self-rated health (t[149]=0.62, p=.266), and gender (χ^2[1]=1.81, p=.179). The groups significantly differed in age (t[149]=-1.73, p=.043), self-rated financial status (t[149]=1.93, p=.028), education level

(χ^2[1]=11.58, p<.001), relationship status (χ^2[1]=10.99, p<.001), and having children (χ^2[1]=18.87, p<.001).

In addition, the groups did not differ in positive affect (t[149]=0.55, p=.291). The groups significantly differed in loneliness (t[149]=-1.87, p=.032), negative affect (t[149]=-1.82, p=.035), and happiness (t[149]=2.30, p=.011).
